# Comparison of survival prediction models for pancreatic cancer: Cox model versus machine learning models

**DOI:** 10.5808/gi.22036

**Published:** 2022-06-30

**Authors:** Hyunsuk Kim, Taesung Park, Jinyoung Jang, Seungyeoun Lee

**Affiliations:** 1 Department of Statistics, University of California, Berkeley, CA 94720, USA; 2 Department of Statistics, Seoul National University, Seoul 08826, Korea; 3 Department of Surgery and Cancer Research Institute, Seoul National University College of Medicine, Seoul 03080, Korea; 4 Department of Mathematics and Statistics, Sejong University, Seoul 05006, Korea

**Keywords:** Cox model, random survival forests, support vector machines, survival prediction model

## Abstract

A survival prediction model has recently been developed to evaluate the prognosis of resected nonmetastatic pancreatic ductal adenocarcinoma based on a Cox model using two nationwide databases: Surveillance, Epidemiology and End Results (SEER) and Korea Tumor Registry System-Biliary Pancreas (KOTUS-BP). In this study, we applied two machine learning methods—random survival forests (RSF) and support vector machines (SVM)—for survival analysis and compared their prediction performance using the SEER and KOTUS-BP datasets. Three schemes were used for model development and evaluation. First, we utilized data from SEER for model development and used data from KOTUS-BP for external evaluation. Second, these two datasets were swapped by taking data from KOTUS-BP for model development and data from SEER for external evaluation. Finally, we mixed these two datasets half and half and utilized the mixed datasets for model development and validation. We used 9,624 patients from SEER and 3,281 patients from KOTUS-BP to construct a prediction model with seven covariates: age, sex, histologic differentiation, adjuvant treatment, resection margin status, and the American Joint Committee on Cancer 8th edition T-stage and N-stage. Comparing the three schemes, the performance of the Cox model, RSF, and SVM was better when using the mixed datasets than when using the unmixed datasets. When using the mixed datasets, the C-index, 1-year, 2-year, and 3-year time-dependent areas under the curve for the Cox model were 0.644, 0.698, 0.680, and 0.687, respectively. The Cox model performed slightly better than RSF and SVM.

## Introduction

Pancreatic cancer is well-known as one of the most lethal cancers worldwide because it has a 5-year overall survival rate of 12.6% as of 2020, while other cancers have 5-year overall survival rates of over 80%. The survival rate strongly depends on the stage of cancer and disease severity. For example, in patients with stage I pancreatic cancer, the 5-year postoperative survival rate is 70.32%, while in patients with stage IV cancer, the 5-year postoperative survival rate is only 3.52%. Therefore, early diagnosis and prediction have been considered promising ways to improve the survival rate of pancreatic cancer.

A survival prediction model for resected pancreatic ductal adenocarcinoma (PDAC) was recently developed with data from the Surveillance, Epidemiology and End Results (SEER) database from the United States and external validation using the nationwide Korea Tumor Registry System-Biliary Pancreas (KOTUS-BP) dataset [1]. This prediction model uses a Cox model, which assumes linear associations with many clinicopathologic features. However, it is necessary to investigate nonlinear relationships or complex interactions between clinicopathologic features associated with the survival time. To this end, we applied two machine learning (ML) methods—random survival forests (RSF) and support vector machines (SVM)—to construct predictive models for survival analysis.

In this study, three different schemes were conducted for model development and external evaluation. First, we utilized data from SEER for model development and data from KOTUS-BP for external evaluation. Secondly, these two datasets were used in reverse by taking data from KOTUS-BP for model development and data from SEER for external evaluation. Finally, we mixed these two datasets half and half and utilized the mixed datasets for model development and external validation.

For each of the three different schemes, we developed prediction models using a Cox proportional hazards model, RSF, and SVM. We compared their performance in terms of the C-index and 1-year, 2-year, and 3-year time-dependent areas under the curve (AUCs).

## Methods

### Data

This study utilized two nationwide databases: the SEER database from the United States and the KOTUS-BP database from Korea. The datasets were pre-processed as described elsewhere [1]. In the screening process, 9,624 patients from SEER and 3,281 patients from KOTUS-BP were selected. Due to the different sets of covariates in the two datasets, only seven covariates—including age, sex, histologic differentiation, adjuvant treatment, resection margin status, and the American Joint Committee on Cancer (AJCC) 8th edition T-stage and N-stage—were utilized in this study.

The SEER database, which has been maintained by the National Cancer Institute in the United States since 1975, is one of the largest and highest-quality cohort studies, whereas the KOTUS-BP database was launched by the Korean Association of Hepato-Biliary-Pancreatic Surgery in 2014 and has been prospectively registered and regularly managed by pancreatobiliary surgeons at specialized centers in Korea. To unify the study period, patients who underwent upfront curative-intent pancreatectomy between 2004 and 2016 were included.

### Model development scheme

Three schemes for model development and external validation were conducted. First, we utilized data from SEER for model development and data from KOTUS-BP for validation. Second, we swapped the roles of these two datasets for model development and evaluation. Finally, we mixed these two datasets half and half, and utilized the mixed datasets for model development and external validation.

**Fig. 1.**
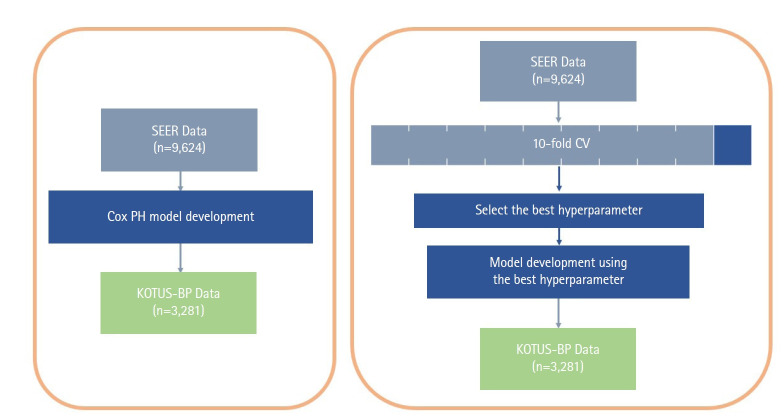
Flowchart of model development and external validation process for the Cox, random survival forests, and support vector machines models. SEER, Surveillance, Epidemiology and End Results; Cox PH, Cox proportional hazard; KOTUS-BP, Korea Tumor Registry System-Biliary Pancreas; CV, cross-validation.

The Cox proportional hazard (Cox-PH) model and the two ML models had different schemes for the model development process, as shown in Fig. 1, although we used all seven covariates in the Cox-PH model and both ML survival models. While the Cox-PH model was constructed without considering any hyperparameters, both RSF and SVM models require cross-validation (CV) to select the set of hyperparameters that build the best model. First, the model development dataset was divided into 10 subsets. For 10-fold CV, nine of the 10 subsets were used for the training set and the other subset was used for the validation set. The average Harrell C-index of the validation sets in a total of 10 iterations was calculated to compare the performance of models. The final model was then constructed with the entire model development dataset and the set of hyperparameters that resulted in the best average Harrell C-index during 10-fold CV.

### ML methods for survival analysis

In prospective cohort studies, survival analysis has been useful to investigate the prognostic factors associated with the survival time and to predict disease processes. In traditional survival analysis, a survival prediction model has been constructed on the basis of demographic and clinicopathologic information. In recent years, there has been considerable interest in applying ML methods to predict the survival of cancer patients using a considerable amount of genomic information including traditional clinical covariates. An advantage of ML methods over the classical Cox regression models is their ability to model complicated associations between the survival time and risk factors, leading to better prediction. Unlike regression and classification settings, standard ML methods cannot be directly applied to censored survival data. With consideration of the censoring mechanism, several ML methods have been extended to survival data, such as bagging survival trees [2], RSF [3,4], SVM for survival analysis [5-8], and CoxBoosting [9]. Among these methods, RSF and SVM for survival analysis were used to develop prediction models for PDAC patients in this study.

### Random survival forests

The RSF method is an extension of Breiman’s random forest method to right-censored survival data by using a forest of survival trees for prediction. Similar to regression and classification settings, RSF is an ensemble learner formed by averaging a tree base-learner. In survival settings, a binary survival tree is the base-learner, and the ensemble learner is formed by averaging each tree’s Nelson-Aalen’s cumulative hazard function.

There are four main steps in RSF: (1) Draw *B* bootstrap samples randomly from the given dataset. Since one-third of the training set data is not present in the bootstrapping sample, this leftover data is known as the out-of-bag (OOB) data. (2) For each sample, construct a survival tree using a randomly selected subset of variables among all available variables, and split the nodes using the candidate variables that maximize the survival difference between child nodes. Here, the survival difference is measured by three criteria: the log-rank statistic, gradient-based Breier score, and log-rank score. (3) Grow the tree to the full size with the constraint that a terminal node should contain a certain number of unique uncensored patients. (4) For each terminal node, calculate the cumulative hazard function (CHF) based on the Nelson-Aalen estimator and take the ensemble CHF of the OOB data by averaging the CHF of each tree.

### SVM for survival analysis

The SVM method of supervised learning has been very successful, mostly in classification and then extended to the regression problem. The main idea of SVM is to minimize the *ε*-insensitive loss function, max(0,|*f*(*x_i_*)-*y_i_* |-*ε*), with a regularization parameter. Here, *f*(*x_i_*), *y_i_*, and *ε* are the predicted value, the actual value, and the acceptable margin of error, respectively.

To take into account censored survival data, SVM for regression on the censored data (SVCR) has been proposed by imposing constraints on the SVM formulation for two comparable cases [5,6]. In other words, for censored data, the time to event after censoring is unknown and thus predictions greater than the censoring time are not required to be penalized. However, all survival predictions less than the censoring time are penalized, while uncensored data are treated in the same way as in the ordinary regression approach, since the exact event time is known. A prior study compared three types of SVM [8], including a regression approach, a ranking approach and a hybrid approach combining the regression and ranking approaches. All types of SVM share a common frame, but they differ in their objective function and constraints. In this paper, two types of SVM were considered: the SVCR model proposed by Shivaswamy et al. [5] and ranking support vector machines (RankSVMs) proposed by Van Belle et al. [8]. These two models have been summarized in detail and compared elsewhere [7].

In the SVM model, overall survival time, y, is explained by the clinical variables x as y=φ(x)+ϵ, where φ(∙) is called the feature map. Since the feature map usually implies a higher-dimensional space, it is unusual to calculate the feature map itself. Instead, the feature map is directly calculated by kernel k(*x_i_*, *x_j_*)=*φ*(*x_i_*)^*T*^ *φ*(*x_j_*) for variable *x* between patients *i* and *j*, which is a consequence of Mercer’s theorem [10]. The entire process of training the model and generating predictions is simply carried out by using the kernel. The kernel plays a significant role in constructing SVM models, and various types of kernels are available, among which a linear kernel and a clinical kernel were considered. The linear kernel is given as $k(x_{i},\;x_{j})\;=\;x_{i}^{T}x_{j}$, whereas the clinical kernel proposed by Daemen and De Moor [11] is defined as the average of the kernel functions, *k*(*x_i_*,*x_j_*), of all variables between patients *i* and *j*. Here $k(x_{i},\;x_{j})\;=\;\frac{(max-min)-\left|x_{i}-x_{j}\right|}{max-min}$ for continuous and ordinal clinical variables and as $k(x_{i},\;x_{j})\;=\;\left\{\begin{array}{l} 1,\;if\;x_{i}\;=\;x_{j}\;\\ 0,\;if\;x_{i}\;\neq \;x_{j} \end{array}\right.$ for nominal clinical variables. The examples presented by Daemen and De Moor [11] show that this kernel better accounts for clinical data, which often have different scales in covariates, and the differences in values of nominal variables are not necessarily linear. The final kernel for clinical data is then the sum of the individual kernel matrices divided by the total number of clinical variables. This final kernel describes the similarity of a class of patients based on a set of variables of different types.

Although SVCR and RankSVMs share the same framework to a certain extent, they differ in terms of how they utilize information for their ultimate objective. SVCR is designed to directly predict the survival time and to minimize the absolute error between predicted and observed survival times. In contrast, RankSVMs focuses on predicting the correct ranking of survival times rather than predicting the actual survival time. In this respect, SVCR extends the standard support vector regression to censored data by penalizing incorrect predictions of censored observations [5,6], while RankSVMs takes into account the ranking problem for the censored data by minimizing the empirical risk of incorrectly ranking two observations.

### Survival prediction models

All statistical analyses were done using R version 3.6.2 (The R Foundation for Statistical Computing, Vienna, Austria). The only continuous covariable, age, was reported as the mean ± standard deviation, and the other categorical variables were reported as frequencies with percentages, as shown in Table 1.

**Fig. 2.**
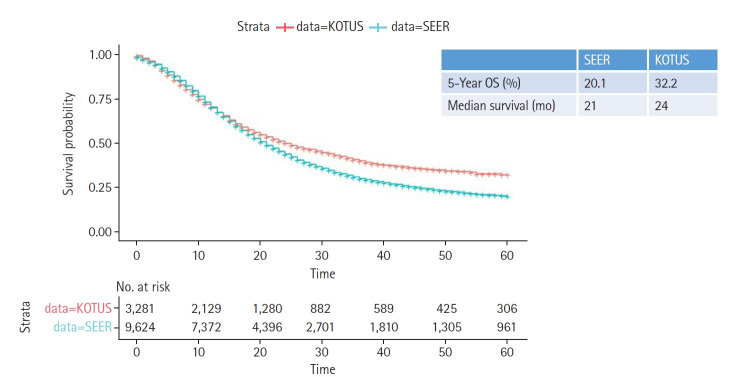
Kaplan-Meier survival curves with 5-year overall survival (OS) rates and median survival times for the Surveillance, Epidemiology and End Results (SEER) and Korea Tumor Registry System-Biliary Pancreas (KOTUS-BP) datasets.

**Fig. 3.**
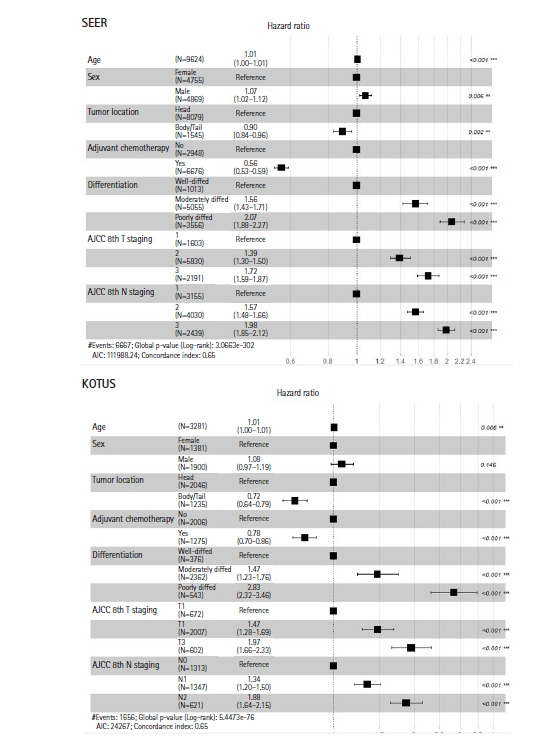
Hazard ratios and 95% confidence intervals of seven variables in the Surveillance, Epidemiology and End Results (SEER) and Korea Tumor Registry System-Biliary Pancreas (KOTUS-BP) datasets. AJCC, American Joint Committee on Cancer.

Two Kaplan-Meier survival curves were compared using the log-rank test, as shown in Fig. 2. In addition, 5-year survival rates and median survival times were given. Variables with p-values less than 0.05 in the univariate Cox model were entered into a multivariate Cox proportional hazards model to estimate the hazard ratios (HRs) for the corresponding predictors, as shown in Fig. 3.

For the implementation of RSF, the number of binary decision trees, the maximum variables for splitting in each node, and the splitting rules for measuring survival differences are shown in Table 2.

The number of trees was 50, 100, 200, 500, and 1,000, and the variables for splitting were given as 10. Although there were seven variables, three variables (histologic differentiation, AJCC 8th edition T-stage, and N-stage) had one more additional variable after one-hot encoding. Three different split rules were applied: log-rank splitting [12,13], gradient-based Brier score splitting [14], and log-rank score splitting [15]. As a result, 150 models for each dataset were constructed, consisting of a combination of the number of trees, the number of variables for splitting, and the split rules.

To implement SVM, 80 models were considered from combinations of various hyperparameters: two SVM models (SVCR and RankSVMs), two types of kernels (linear and clinical kernels), two ways of computing distance between data points (makediff1 and makediff3), and 10 values of the regularization parameter γ as shown in Table 3. The two arguments makediff1 and makediff3 are used in the R package *survivalsvm*, in which makediff1 computes the distance between two consecutive observations only when the first one is not censored, whereas makediff3 computes the difference between data point *i* and its neighbor that has the largest survival time that is smaller than the survival time of y_*i* [8]. In total, 80 models were cross-validated and the model with the best validation C-index was chosen.

### Advantages of ML methods over the Cox model

Based on three survival predictive models, we investigated personalized treatment policies using the survival rate over time. It is well known that the Cox model assumes a proportional HR over time, which implies that the HRs between different individuals are constant over time. However, the two ML models used in this study reflect more complex interactions between covariates and yield non-constant HRs between different individuals over time. For personalized treatment, it would be more desirable to predict the survival rate over time using the ML models than using the Cox model.

## Results

Fig. 2 shows the two Kaplan-Meier survival curves for the SEER and KOTUS-BP datasets. The censoring fractions were 30.7% for SEER and 49.5% for KOTUS-BP. These two survival curves overlapped for up to 20 months and then significantly separated, with a p-value less than 1e-13 from the log-rank test for the equivalence of two survival curves. The median survival times were 21 months for SEER and 24 months for KOTUS-BP. Fig. 3 shows the estimated HRs of each clinical variable from the multivariate Cox model with 95% confidence intervals and p-values for both datasets. The estimated HRs of the seven variables were not meaningfully different between the datasets, and all the variables were significant, except for sex in KOTUS-BP dataset.

Table 4 shows the C-index values and 1-year, 2-year, and 3-year time-dependent AUCs for the final Cox, RSF, and SVM survival analysis models according to the three schemes of model development. The Cox, RSF, and SVM models performed somewhat better when the two datasets were mixed half and half than when the first and second schemes were applied. Although the results are not shown here, the performance of RankSVMs was exceptionally low, with C-index values of 0.548, 0.529, and 0.514 for the three schemes, respectively. This implies that the rank-based approach performed worse than the regression-based approach in this study.

Through 10-fold CV of 150 RSF models, the model with the best validation Harrell C-index was chosen as the final RSF model. The final RSF model consisted of 100 decision trees, used a maximum of two variables in splitting nodes, and used the log-rank test to measure survival differences when two datasets were mixed half and half. The C-index and 1-year, 2-year, and 3-year time-dependent AUCs were 0.6337, 0.6824, 0.6681, and 0.6781, respectively.

Similarly, the model with the best validation Harrell C-index was chosen through 10-fold CV of 80 SVM models. The final SVM model was the SVCR model based on an additive clinical kernel, regularization constant (γ) of 0.1, and the makediff3 method to calculate the distance between data points when the two datasets were mixed half and half. The C-index and 1-year, 2-year and 3-year time-dependent AUCs were 0.6233, 0.6849, 0.6352, and 0.6264, respectively.

The C-index and 1-year, 2-year, and 3-year time-dependent AUCs of the Cox model were 0.6434, 0.6976, 0.6795 and 0.6873, respectively. Comparing these values to those of the two ML survival models, the Cox model consistently performed slightly better than RSF and SVM models. The Cox model also yielded slightly better results when the two datasets were mixed half and half than when the two datasets were not mixed.

**Fig. 4.**
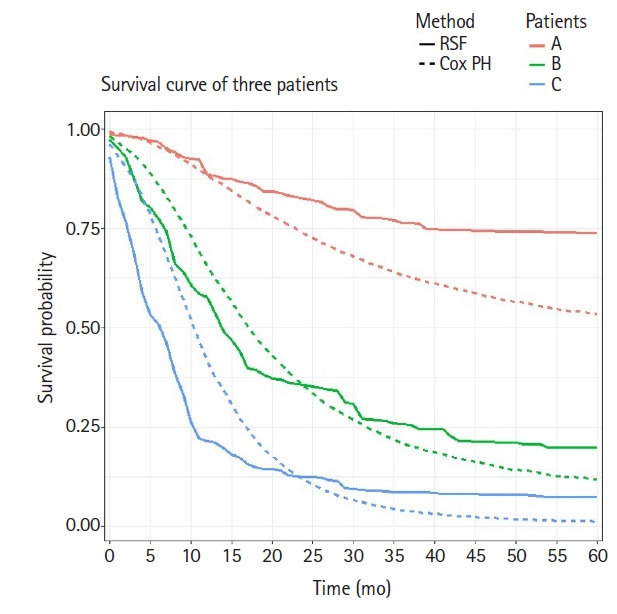
Overlaid predicted survival curves of the Cox model and random survival forests (RSF) method for three patients. Cox PH, Cox proportional hazard.

In order to consider personalized treatment policies, we compared the predictive survival curves of three different patients using the fitted Cox model and the final RSF model described above. Suppose that the three chosen patients (A, B, and C) are all 50-year-old women, have a tumor in the body or tail of the pancreas, and have not received chemotherapy. Patient A has a well differentiated tumor staged T1 and N0 according to the AJCC 8th edition staging system. Patient B has a moderately differentiated tumor staged T2 and N1, whereas patient C has a poorly differentiated tumor staged T3 and N2. We plotted two predicted survival curves from both the Cox model and RSF model for these three patients over time, as shown in Fig. 4. The predicted survival curves from the Cox model showed relatively consistent differences among these three patients over time, whereas those from the RSF model showed less consistent differences. For example, the slope of the survival curve of patient C suddenly changed at 10 months after the diagnosis and the slope of patient B dramatically changed at 17 months after the diagnosis, whereas the slope of patient A did not change over time. Therefore, it seems that there is a discrepancy between the survival curves generated using the Cox and RSF models. This may imply that different treatment strategies for different patients would maximize treatment efficacy.

## Discussion

In light of the development of a predictive survival model for PDAC [1], we considered a comparative study to investigate whether ML methods for survival analysis improve the predictability of the survival rate. In this study, both RSF and SVM methods for survival analysis were considered and compared with the Cox model [1] using the same SEER and KOTUS-BP datasets. In addition to the scheme used in the previous model [1], two other schemes were considered for model development and evaluation. In the second scheme, the roles of these two datasets were reversed, so that KOTUS-BP was used as the training set and SEER was used as the external validation set. In the third scheme, these two datasets were mixed half and half, and one of the mixed datasets was randomly chosen for model development and the remaining dataset was used for external validation. As shown in the Results section, the third scheme yielded slightly better performance for all methods than the other two schemes.

Compared with the Cox model, the performance of the ML survival models was not significantly improved, and RSF performed similarly to the Cox model. However, the performance of SVM differed substantially according to how the survival information was used. The performance of SVCR was comparable to those of the Cox model and RSF, since SVCR utilizes the survival time in the regression model considering the censoring mechanism. In contrast, the performance of RankSVMs was not good because this method only uses the ranking information of the survival times.

The RSF and SVM showed no substantial improvements in performance compared to the Cox model. In this study, only seven clinical variables were shared between the SEER and KOTUS-BP datasets, which might have been too few to maximize the usefulness of ML methods. ML methods are useful to analyze more complex and nonlinear associations among high-dimensional variables such as genetic information. It was also noted that the Harrell C-index of all models, both in the training set and in the test set, was less than 0.70, except for one or two cases.

Although it takes more time to develop ML survival models than a Cox model and there is no substantial performance improvement, these ML survival models have the advantage of allowing nonlinear risk to be predicted over time. As shown in Fig. 4, the trend in the survival curves for the RSF model was different from that for the Cox model. For example, the survival curves of the RSF model had the largest difference between patients B and C when 1 year to 2 years had passed. With this information, clinicians can pay particular attention to patient C in this period. Meanwhile, patients in a period with particularly high risk can be informed in advance, so that they could receive additional health care in that period. The fact that the RSF model outputs the survival curve of each individual might enable more patient-specific care. Furthermore, ML survival models recommend whether a patient should receive treatment or not [16]. Adjuvant chemotherapy was found to be helpful for almost all of the patients in this study, but other treatments can be rather harmful to some patients.

## Figures and Tables

**Table 1.** Basic statistics and 5-year overall survival rates for seven variables in the SEER and KOTUS-BP databases

Variable	SEER database (n=9,624)			KOTUS database (n=3,281)		
	Patients	5-Year OS (%)	p-value^a^	Patients	5-Year OS (%)	p-value^a^
Age (yr)	65.6±10.4	20.1		63.8±10.1	32.2	
Female	4,755 (49.4)	21.3		1,381 (42.1)	36.2	
Male	4,869 (50.6)	18.9	0.006	1,900 (57.9)	29.2	0.146
Head	8,079 (83.9)	19.2		2,046 (62.4)	28.5	
Body/Tail	1,545 (16.1)	25.0	0.002	1,235 (37.6)	37.8	<0.001
No adjuvant treatment	2,948 (30.6)	17.3		2,006 (61.1)	29.5	
Adjuvant treatment	6,676 (69.4)	21.3	<0.001	1,275 (38.9)	36.1	<0.001
Well differentiated	1,013 (10.5)	37.4		376 (11.5)	44.9	
Moderately differentiated	5,055 (52.5)	20.5	<0.001	2,362 (72.0)	32.9	<0.001
Poorly differentiated	3,556 (37.0)	14.6	<0.001	543 (16.5)	20.8	<0.001
T1	1,603 (16.7)	32.7		672 (20.5)	45.3	
T2	5,830 (60.6)	18.8	<0.001	2,007 (61.2)	29.7	<0.001
T3	2,191 (22.7)	14.3	<0.001	602 (18.3)	24.5	<0.001
N0	3,155 (32.8)	32.4		1,313 (40.0)	42.6	
N1	4,030 (41.9)	20.5	<0.001	1,347 (41.1)	28.5	<0.001
N2	2,439 (25.3)	14.6	<0.001	621 (18.9)	16.4	<0.001

Values are presented as mean±SD or number (%). SEER, Surveillance, Epidemiology and End Results; KOTUS-BP, Korea Tumor Registry System-Biliary Pancreas; OS, overall survival.

a Log-rank test.

**Table 2.** Hyperparameters for random survival forests

Hyperparameter	Value
No. of trees	50, 100, 200, 500, 1,000
Max. variables used in split	1‒10
Splitting rule	log-rank/bs.gradient/logrankscore

One-hot encoded variables: differentiation, AJCC 8th edition T and N staging. AJCC, American Joint Committee on Cancer.

**Table 3.** Hyperparameters for support vector machines for survival analysis

Hyperparameter	Value
SVM type	SVRC, RankSVMs
Kernel	Linear, clinical
Distance matrix	Makediff1, makediff3
Regularization constant	0.01, 0.02, 0.05, 0.1, 0.2, 0.5, 1, 2, 5, 10

SVM, support vector machines; SVRC, support vector regression for censored data.

**Table 4.** C-index and 1-year, 2-year, 3-year time-dependent AUCs for the Cox, RSF, and SVM models according to three schemes

Model	Training				Test			
	C-index	Td1 AUC	Td2 AUC	Td3 AUC	C-index	Td1 AUC	Td2 AUC	Td3 AUC
Training (SEER)					Test (KOTUS)			
Cox	0.65417	0.72545	0.68776	0.68765	0.62792	0.65489	0.66759	0.68153
RSF	0.66520	0.72960	0.70807	0.71722	0.63344	0.66660	0.67675	0.69104
SVM	0.64218	0.72258	0.65812	0.64074	0.59956	0.61514	0.62619	0.63458
Training (KOTUS)					Test (SEER)			
Cox	0.65074	0.69346	0.69524	0.70095	0.62932	0.68365	0.67008	0.67426
RSF	0.66293	0.70624	0.71295	0.71676	0.62189	0.67445	0.65885	0.66058
SVM	0.62668	0.66973	0.66769	0.66072	0.60061	0.64794	0.63057	0.62372
Training (SEER + KOTUS)					Test (SEER + KOTUS)			
Cox	0.64890	0.70718	0.69108	0.69327	0.64361	0.69764	0.67953	0.68726
RSF	0.66396	0.71328	0.72110	0.73110	0.63363	0.68239	0.66810	0.67806
SVM	0.62538	0.69700	0.64029	0.61994	0.62333	0.68489	0.63515	0.62643

AUC, area under receiver operating characteristic curve; RSF, random survival forests; SVM, support vector machines; C-index, Harrell’s concordance index; Td1, 1-year time-dependent; Td2, 2-year time-dependent; Td3, 3-year time-dependent; KoTUS, Korea Tumor Registry System-Biliary Pancreas; SEER, Surveillance, Epidemiology and End Results.
